# Supplementary material for: Smart and Age-Friendly Cities in Russia: An Exploratory Study of Attitudes, Perceptions, Quality of Life and Health Information Needs
Source: Int J Environ Res Public Health. 2020 Dec 9;17(24):9212. doi: 10.3390/ijerph17249212 (PMC7764761; doi:10.3390/ijerph17249212)
Supplement: Supplementary file 1 [file ijerph-17-09212-s001.zip › Supplementary Material S1 English translation of the survey questionnaire.pdf]

Appendix S1

English translation of the survey questionnaire "Health evidence for consumers"

Dear participant,

We invite you to take part in the survey on important health matters and the use of evidence in medicine and health care.

This is truly critical now during the COVID-19 pandemic.

This survey is being conducted within the framework of the project "Evidence School for ageing population of Tatarstan to improve health and longevity through Cochrane Knowledge Translation", supported by a grant from Cochrane and conducted Cochrane Russia team (http://russia.cochrane.org) with the participation of groups of experts and consultants from medical and educational institutions of Kazan.

The survey results will help us to understand the problems of ageing population in health and healthcare, their needs, and will be used to develop resources for the program of Evidence School and further development in this area.

Section I - Participant portrait

Please indicate your age: exactly ____ or using these ranges

☐ under 60 years old
☐ 60 - 70 years old
☐ 70 - 80 years old
☐ over 80 years old

Your gender:

☐ Female
☐ Male

Your education:

☐ Secondary (high-school)
☐ Secondary special (technical school, college, etc.) (except for medical / pharmaceutical)
☐ Secondary specialized medical / pharmaceutical
☐ Higher (except for medical / pharmaceutical)
☐ Higher medical / pharmaceutical

Occupation:

☐ Medicine, health care, pharmacy
☐ Another professional field
☐ Not employed

Please indicate who you are at the time of the survey

☐ I am a patient at the ICDC
☐ I am a caring relative / acquaintance of a patient at ICDC
☐ I am an employee of the ICDC
☐ Your own version: ____

What chronic diseases do you have? (indicate all you know)

☐ Cardiovascular diseases
☐ Respiratory diseases of system (bronchitis, asthma)
☐ Gastrointestinal diseases (gastritis, duodenitis, ulcer, colitis and others)
☐ Musculoskeletal diseases (arthritis, arthrosis)
☐ Neurological diseases

☐ Hepato-biliary diseases
☐ Diseases of the kidneys and bladder
☐ Endocrine diseases (diabetes mellitus, hypothyroidism, hyperthyroidism and others)
☐ Diseases of the female genital area
☐ Diseases of the male genital area
☐ Your own answer ___________________________________

Do you have any bad habits? (specify all if any)
☐ Smoking
☐ Excess alcohol consumption
☐ Excess nutrition
☐ Sedentary lifestyle
☐ Disorderly lifestyle
☐ No attention to your health
☐ Your own answer ___________________________________

How often do you take medicines?
☐ Daily
☐ From time to time
☐ Rarely
☐ Never
☐ Your own answer: ___________________________________

What medicines do you take continuously or for a long time (for chronic or long-term diseases)? (please indicate if you take)

____

Do you use non-drug treatment (physical therapy, physiotherapy, massage, psychotherapy, behavioural therapy, etc.)?
☐ Yes, I use
☐ No, I don't use
☐ Your own answer: ___________________________________

Section II – Awareness level

1. Have you heard about evidence-based medicine before?
☐ yes
☐ no
☐ find it difficult to answer

a. If yes, then, what is the evidence-based medicine, in your opinion?

____

2. Have you ever heard about Cochrane and the work of this Organisation?
☐ yes
☐ no
☐ find it difficult to answer

a. If yes, then, how could you briefly define Cochrane, the vision and mission of the organisation?

____

b. If no, then would you like to learn more about evidence-based medicine and Cochrane (for example, to take part in Cochrane Evidence School)?
☐ yes
☐ no
☐ find it difficult to answer

3. Have you ever heard about ageism?
☐ yes
☐ no
☐ find it difficult to answer

a. If yes, how would you describe what ageism is?

____

4. Do you think that treatment and prevention methods, which are currently used in healthcare, have been proven to be effective and safe in high-quality rigorous research?
☐ yes
☐ not
☐ find it difficult to answer

a. If yes, could you define what you consider to be quality research?

____

b. If no, what do you see as the reason for using technologies that have not been proven by rigorous research to be effective?

____

Section III - Exploring aging / health problems

5. Has it happened and does it happen that you take medicines or undergo a diagnostic procedure without a doctor's recommendation?
☐ yes
☐ no
☐ I find it difficult to answer

a. If yes, what medicines, or what methods of diagnostics you use (d)?

____

6. Are you trying to independently obtain information you are interested in your health, diseases (if any), methods of health promotion, treatment and prevention?
☐ yes
☐ no
☐ find it difficult to answer

a. If yes, then where do you look for information and where do you find it?

____

b. If not, then what information do you trust and rely on?

____

7. Have you encountered any problems related to your age?
☐ yes
☐ no

 find it difficult to answer

a. If yes, could you describe these problems?

____

8. How do you rate your quality of life on a scale from 1 to 10 (1 - poor, 10 - excellent)?
1 2 3 4 5 6 7 8 9 10

9. Has your quality of life changed with age?
 yes
 no
 find it difficult to answer

a. If yes, then how?

____

10. Are you satisfied with your current situation?
 yes
 no
 find it difficult to answer

a. If not, what would you like to change?

____

11. Have you ever taken part in anti-aging programs?
 yes
 no
 find it difficult to answer

a. If yes, how do you see the benefits?

____

b. If no, then why?

____

12. What health matters are you interested in, and would you be interested to learn about them at the Evidence School and / or receive information in future? (more than one answer are possible)
 Questions related to the use of medicines (efficacy, safety) for a disease/condition (indicate the diseases/conditions you are interested in ______________ ________ ____________________)
 Questions related to the use of non-drug treatment (indicate the diseases/conditions you are interested in ______________________________ ____)
 Questions related to prevention of diseases (indicate the diseases you are interested in ____)
 Questions related to diagnostics of diseases
 Other questions (specify which ____)

13. What questions or problems do you face in treating diseases or in your efforts to maintain and strengthen your health ?

____

14. If you are interested in independent, evidence-based information on health and medical matters, including on the current COVID-19 pandemic and its impact on your health and your usual therapy, in what form and in what way would it be convenient for you to receive

this information ?
(or suggest answers)

Thank you for participating in the survey!
Take care of yourself and be healthy!
