# Supplementary material for: Smart and Age-Friendly Cities in Russia: An Exploratory Study of Attitudes, Perceptions, Quality of Life and Health Information Needs
Source: Int J Environ Res Public Health. 2020 Dec 9;17(24):9212. doi: 10.3390/ijerph17249212 (PMC7764761; doi:10.3390/ijerph17249212)
Supplement: Supplementary file 1 [file ijerph-17-09212-s001.zip › Supplementary Material S2 Informed consent form.pdf]

Informed consent form for participation in a survey

The survey is carried out within the framework of the project "Cochrane Evidence School for the population of the Republic of Tatarstan to improve health and longevity through the Broadcast of Cochrane Knowledge", supported by a Cochrane grant and conducted by Cochrane Russia community (http://russia.cochrane.org) with the participation of a group of specialists and consultants from medical and educational institutions of Kazan.

The results of the survey will make it possible to understand the most significant issues and problems in the field of health and medicine use for the older population, their needs, and will be used to develop evidence products within the framework of the Cochrane Evidence School and further development in this area.

I received and understood the information about the research project and I agree:

- to participate in the survey
- to provide my personal demographic information and its anonymized processing.

Date

Signature
